# Metacognitive Monitoring and Control of Eyewitness Memory Reports in Autism

**DOI:** 10.1002/aur.2278

**Published:** 2020-02-13

**Authors:** Katie Maras, Jade Eloise Norris, Neil Brewer

**Affiliations:** ^1^ Centre for Applied Autism Research University of Bath Bath UK; ^2^ Flinders University Adelaide Australia

**Keywords:** autism, memory, interviewing, support, witness, grain size, metacognition, monitoring

## Abstract

Providing eyewitness testimony involves monitoring one's memory to provide a *detailed* and *accurate* account: reporting details likely to be accurate and withholding potentially inaccurate details. Autistic individuals reportedly experience difficulties in both retrieving episodic memories and monitoring their accuracy, which has important implications for eyewitness testimony. Thirty autistic and 33 IQ‐matched typically developing (TD) participants viewed a video of a mock bank robbery followed by three phases of questions (with judgments of confidence). In Phase 1, participants freely generated the granularity of their responses (i.e., fine‐ or coarse‐grained). In Phase 2, participants answered the same questions but provided both a fine‐ *and* a coarse‐grained answer. In Phase 3, participants were instructed to maximize accuracy over informativeness by selecting one of their Phase 2 answers as their final answer. They either received the questions socially (from the experimenter) or answered them online. There were no group differences in accuracy or metacognitive monitoring, with both autistic and TD witnesses demonstrating: (a) a strong preference for reporting fine‐grained details at the expense of accuracy; (b) improved though still suboptimal grain size reporting when instructed to maximize accuracy over informativeness; (c) effective accuracy monitoring; and (d) higher overall accuracy when questions were delivered socially. There was, however, a subtle difference in metacognitive control, with autistic witnesses performing more poorly than TD witnesses when questions were delivered socially, but not when they were delivered online. These findings contrast with evidence suggesting that autism is marked by impairments in episodic memory and metacognitive monitoring and control. ***Autism Res** 2020, 13*: 2017‐2029. © 2020 The Authors. *Autism Research published by International Society for Autism Research* published by Wiley Periodicals, Inc.

**Lay Summary:**

Autistic people have been reported to experience subtle difficulties in monitoring and regulating their information reporting, which has important implications for providing eyewitness testimony. We found that autistic witnesses' testimony comprised a similar level of detail and accuracy as non‐autistic witnesses' accounts. However, autistic people found it difficult to optimize their testimony when the questions were delivered socially—but not when they answered the questions online. © 2020 The Authors. *Autism Research published by International Society for Autism Research* published by Wiley Periodicals, Inc.

## Introduction

Recalling information from memory involves monitoring output for informativeness (to provide as much detailed information as possible) and accuracy (to avoid reporting incorrect information). According to Koriat and Goldsmith's [1996] monitoring and control framework, when responding to questions, an individual first attempts to retrieve a fine‐grained (FG) (i.e., detailed) response. Confidence in the accuracy of this detail is then evaluated and compared with a criterion value. A typical individual will volunteer a FG response (e.g., “navy blue” in response to “what color was his hat?”) when their confidence exceeds this criterion and if not, the individual attempts to retrieve a less detailed, coarse‐grained (CG) response [e.g., “dark”; Ackerman & Goldsmith, 2008]. This process of memory monitoring and control has important implications for eyewitness testimony, where it is crucial to maximize the amount of information witnesses provide while also ensuring accuracy. Importantly, it also suggests that the compromise between accuracy and informativeness is under the strategic control of the witness, rather than simply a result of memory encoding or maintenance processes [Weber & Brewer, 2008].

There is now a large body of evidence showing that, alongside the core behavioral features of autism, autistic individuals show marked differences in memory processes compared to typically developing (TD) individuals [see e.g., Boucher & Bowler, 2008; Boucher, Mayes, & Bigham, 2012]. In particular, autistic individuals often experience specific difficulties in retrieving episodic memories [see e.g., Crane & Maras, 2018; Gaigg & Bowler, 2018]. A number of studies also report that autistic individuals show difficulties in monitoring the accuracy of their responses [e.g., Brosnan et al., 2016; Grainger, Williams, & Lind, 2014, 2016; Williams, Bergström, & Grainger, 2018; but see Sawyer, Williamson, & Young, 2014, and Wojcik, Allen, Brown, & Souchay, 2011]. Given that monitoring is used to regulate or “control” reporting choices [Koriat & Goldsmith, 1996], it is plausible that difficulties with metacognitive monitoring play a key role in the reported difficulties in autistic people's strategic reporting of episodic information. However, little research to date has examined metacognitive *control* in autism, and none to our knowledge has specifically tested this in the context of memory for episodic events.

Grainger et al. [2016] examined autistic and TD children's monitoring and control processes on a general knowledge task. To test metacognitive monitoring, participants provided “judgments of confidence” (also known as “realism of confidence judgments”) in their answers to questions about recently studied material. To test metacognitive control, participants were told that for each correct answer, they would receive a point, but for each incorrect answer, they would lose a point, and at the end of the task children were able to remove any of their previously provided answers. The autistic group showed evidence of metacognitive monitoring difficulties, with poorer confidence–accuracy calibration and smaller difference scores between their judgment of confidence ratings for correct versus incorrect answers, compared to TD children. The autistic participants also showed reduced use of monitoring to control their reporting, with a significantly smaller mean difference between judgment of confidence ratings for kept and removed answers than TD participants.

Findings from other studies are mixed. Sawyer et al. [2014] used a similar design to examine metacognitive monitoring and control on a facial emotion recognition task and a general knowledge task. Overall, autistic and TD adults did not differ on either task for the measures of metacognitive monitoring (realism of confidence judgments) or control (withholding incorrect responses and volunteering correct responses; *d*′). Nevertheless, a high proportion of autistic participants (40%; vs. 13.5% of TD participants)—notably those who had shown diminished metacognitive monitoring—chose not to withhold any answers at all on the emotion recognition task, indicating potential subtle underlying metacognitive control difficulties [see Grainger et al., 2016, for further discussion].

Maras, Gamble, and Brosnan [2019] tested metacognitive monitoring and control in autistic and TD children on a Mathematics gaming task and also reported no differences between groups in realism of confidence judgments (although the autistic group did show a general bias toward higher confidence). However, the autistic children showed reduced cohesion between their pre‐ and post‐test intentions (e.g., pre‐test they indicated that their intention was to get the answer right, but post‐test they reported that they had meant to get it wrong), again potentially indicating subtle difficulties in monitoring. Furthermore, despite no significant difference between groups on the measure of control (the number of points won), there was a strong trend for reduced strategy use by autistic participants, who averaged around half the number of points of the TD group when no support (such as strategy reminders) was provided. Finally, Cherkaoui and Gilbert [2017] reported that while autistic participants were undiminished in predicting their (poorer) performance on a prospective memory task, they failed to compensate for this with an increased use of reminders during the task. Thus, metacognitive monitoring is necessary but not sufficient for effective strategy regulation; even if autistic individuals' metacognitive monitoring per se is unimpaired, they may nevertheless experience difficulties in *using* monitoring processes to strategically control their reporting [Sheppard, Bruineberg, Kretschmer‐Trendowicz, & Altgassen, 2018].

These mixed findings indicate further investigation of metacognitive monitoring and control in autism is warranted, specifically for the reporting of episodic events (where autistic difficulties are often noted), especially given the often important implications of such reports for both every day and high stakes real‐life situations (e.g., providing eyewitness testimony). Autistic individuals are more likely to have interactions with police as victims/witnesses^1^ and may, therefore, be required to provide a detailed account of an incident [e.g., Brown‐Lavoie, Viecili, & Weiss, 2014; Weiss & Fardella, 2018]. However, evidence suggests that, compared with TD individuals, autistic witnesses often provide testimony that is less accurate and/or less complete [see Maras, 2020; Maras & Bowler, 2014].

Research to date, however, has not considered the level or “grain size” of detail provided by autistic witnesses (e.g., whether the information reported is at the FG or CG level), nor their ability to metacognitively monitor and control their reporting decisions. Thus, we know little about the informativeness of autistic witnesses' memory reports, or about the underlying monitoring and control processes. This is critical because the number of details reported is not necessarily indicative of how informative the testimony is; for example, to rule out all but one of the suspects it may be critical to obtain the exact color of the perpetrator's jacket (e.g., brown), rather than a broader response (e.g., dark). Understanding whether differences in autistic and TD witnesses' accuracy and completeness are the result of differences in monitoring and/or control processes is also crucial for developing appropriate interview support [see Maras, 2020].

In examining performance by autistic people, it is also important to consider how the task is delivered (e.g., socially or online) and whether the underlying task requirements (e.g., to maximize accuracy or informativeness, or both) are explicitly stated. Several researchers have argued that autistic participants' performance on tasks is often under‐estimated because social cognition difficulties hinder their ability to infer the implicit demands of the task [see e.g., Kenworthy, Yerys, Anthony, & Wallace, 2008; White, 2013; White, Burgess, & Hill, 2009]. For example, difficulties that are frequently observed when instructions and questions are administered socially often dissipate when more explicit instructions are provided, or when computerized versions of the task are used [e.g., Chevallier et al., 2014; Ozonoff, 1995]. Socially mediated tasks may also place greater sensory and executive demands on autistic individuals. Hsu and Teoh [2017] examined the impact of socially administered questions on event memory in autism by interviewing autistic and TD children about an event that they had participated in either a traditional face‐to‐face format or via an avatar. There was some evidence that the avatar elicited more information and higher accuracy than the human interviewer for the autistic (but not the TD) children. Hsu and Teoh suggested that the avatar interviewer reduced demands on social processing and minimized potential overloading of the sensory system, allowing autistic children to attend more closely during questioning, which in turn improved their episodic recall. It has also been argued that autistic individuals experience attenuated social motivation and diminished concern for reputation management [see Chevallier, Kohls, Troiani, Brodkin, & Schultz, 2012; but see Jaswal & Akhtar, 2018]. Explicit instructions and computerized task versions may, therefore, “level the playing field” by being less overloading and ambiguous, and equally motivating for both autistic and TD individuals.

TD witnesses, in contrast, often perform better in social contexts. Although TD individuals can and do monitor and control their reporting in terms of accuracy and informativeness, they nevertheless show a tendency to maximize informativeness over accuracy [e.g., Brewer, Vagadia, Hope, & Gabbert, 2018; McCallum, Brewer, & Weber, 2016]. This tendency is attenuated, however, when they are required to read their responses aloud to the experimenter compared to when their responses are obtained in private [McCallum et al., 2016]. It has been suggested that answering in a social context may motivate TD participants to be more accurate to avoid embarrassment by reporting more accurate CG detail than risk providing inaccurate FG information [McCallum et al., 2016; but see Taylor & Dando, 2018]. Furthermore, McCallum, Brewer, and Weber [2019] argue that witnesses construe informativeness based not only on the degree of specificity in their memories but also on their perceptions of the value or utility of the information. Thus, TD individuals are also more likely to report a higher proportion of (more accurate) CG information than they would otherwise report when they are instructed that accuracy should be prioritized over informativeness [e.g., Goldsmith, Koriat, & Weinberg‐Eliezer, 2002; Weber & Brewer, 2008].

The present study examined the role of metacognitive monitoring and control processes in the informativeness and accuracy of autistic and TD witnesses' memory reports, and the impact of task instructions and format (social or online) on this. We predicted that autistic witnesses would show difficulties with metacognitive monitoring and control, and therefore a greater tendency to report FG detail with reduced accuracy compared to TD participants. When the need for accuracy was made explicit, however, it was predicted that both autistic and TD participants' recall accuracy would be improved (driven by a shift from FG to CG responding), somewhat ameliorating the difference between groups. We expected the format of the task to differentially impact each group's performance. Specifically, we expected TD witnesses to perform better when questions were delivered socially, while difficulties with social cognition and diminished motivation would impede autistic participants' performance when questions were delivered socially. We expected similar performance from both groups, however, when questions and responses were computerized.

## Method

### *Participants*

A power analysis using G*Power3.1 [Faul et al., 2007] indicated that a sample size of 60 would give 80% power to detect medium‐to‐large effects of Group, Delivery, and Phase (i.e., to have meaningful implications for practice). A total of 63 participants took part: 30 autistic adults (16 males, 14 females) and 33 TD participants (seven males; 26 females). Participants were recruited mainly from the South West of England and surrounding areas, including via previous research participation, autism‐related and local community Facebook groups, social and support groups, as well as via local community recruitment (including posters, magazine articles, and social media posts, and University website campaigns).

All autistic participants had received a formal clinical diagnosis of Autism Spectrum Disorder according to DSM–IV [American Psychiatric Association, 2000] or DSM‐5 criteria [American Psychiatric Association, 2013], and confirmed this with a copy of their clinical diagnostic report (NB. a breakdown of scores from the ADOS was only available for three participants). Six participants had received a clinical diagnosis but were unable to access their report and were therefore administered the Autism Diagnostic Observation Schedule, Second Edition [ADOS‐2; Lord et al., 2012] to confirm their diagnoses. The total ADOS scores for the nine participants who had scores available were as follows: Communication *M* = 3.00, *SD* = 1.41, range = 1–6; Reciprocal Social Interaction *M* = 7.56, *SD* = 1.74, range = 6–11.

Autistic and TD participants were randomly assigned to complete the study in either a social delivery condition (where questions were delivered by the experimenter) or online. There were no effects of Group, Delivery, or Group × Delivery interactions for age (all *P*s > 0.392, *ηP* ^2^s < 0.01), or on measures from the Wechsler Abbreviated Scale of Intelligence‐Second Edition [WASI‐II; Wechsler, 2011]: Verbal Comprehension Index (all *P*s > 0.353, *ηP* ^2^s < 0.02), Perceptual Reasoning Index (all *P*s > 0.124, *ηP* ^2^s < 0.04), and full‐scale IQ (all *P*s > 0.136, *η*P^2^s < 0.04). All non‐autistic participants scored below the recommended minimum cut‐off of 32 on the Autism Spectrum Quotient [AQ‐50, Baron‐Cohen, Wheelwright, Skinner, Martin, & Clubley, 2001] and, as expected, the autistic group scored significantly higher on the AQ than TD participants (*P* < 0.001, *η*P^2^ = 0.71), but there was no main effect of Delivery (*P* = 0.666, *η*P^2^ < 0.01), or Group × Delivery interaction (*P* = 0.337, *η*P^2^ = 0.02) (see Table 1). Participants provided their written informed consent to take part and were fully debriefed. Ethical approval was obtained from the Psychology Research Ethics Committee at the University of Bath.

**Table 1:** Mean and Range Age, IQ, and AQ‐50 Scores for Autistic and TD Participants Within Each Delivery Condition (*SD*s are in parentheses)

	TD		Autistic	
	Social (*n* = 15)	Online (*n* = 15)	Social (*n* = 18)	Online (*n* = 15)
Age (years)	33.67 (10.93); range 18–51	35.93 (12.63); range 19–60	34.53 (14.55); range 18–59	31.33 (12.14); range 19–62
Verbal Comprehension Index	110.33 (5.57); range 102–125	107.00 (11.44); range 79–123	106.40 (9.33); range 90–121	106.27 (12.44); range 85–128
Perceptual Reasoning Index	112.11 (11.89); range 93–136	110.40 (11.35); range 92–135	108.73 (14.47); range 82–131	103.73 (13.24); range (82–128)
Full‐scale IQ	112.67 (7.60); range 101–125	109.80 (10.67); range 88–132	108.53 (12.05); range 89–129	105.67 (12.86); range 86–132
AQ‐50	12.94 (6.89); range 1–24	10.20 (5.28); range 2–17	34.29 (10.12); range 14–46	35.33 (7.94); range 21–46
Gender	3 males, 15 females	4 males, 11 females	8 males, 7 females	8 males, 7 females

### *Materials*

#### *Stimulus video*

Participants viewed a short video clip of a mock bank robbery lasting 36 s. The clip showed two males taking cash from a female bank teller before running from the bank and leaving in a getaway car driven by an accomplice [see also McCallum et al., 2016].

#### *Recall questions*

The recall questionnaire comprised 20 questions developed by McCallum et al. [2016] and required either color, numerical, or time‐based answers (e.g., the color of the suspects' clothing, number of witnesses, age of the getaway car, duration of robbery, etc.; see Appendices 1–3). Following each question, participants were asked to estimate their confidence that their answer was correct on a sliding scale with 10% increments (i.e., 0%–100%).

### *Design and Procedure*

The study used a 2 (Group: autistic, TD) × 2 (Delivery: social, online) × 3 (Phase: Phase 1 *free report*, Phase 2 *forced report*, Phase 3 *instructions to maximize accuracy*) mixed design, where Phase was within participants. Participants were tested individually. They were informed that they were about to view a stimulus video (alone and in a separate room) and that they would be asked some questions about it afterward. Half of the participants received the questions socially (by the experimenter); the other half completed the questions online in a separate room. In the social condition, a female experimenter provided instructions for the task and presented the questions. Following Chevallier et al. [2014], in the non‐social condition, both task instructions and the test questions were presented online, with the experimenter not present in the room. Approximate task duration did not differ between Delivery conditions, *F*(1,59) = 1.37, *P* = 0.247, *ηP* ^2^ = 0.02, or Group, *F*(1,59) = 0.22, *P* = 0.643, *ηP* ^2^ = 0.01, and there was no significant Delivery × Group interaction = *F*(1,59) = 1.21, *P* = 0.277, *ηP* ^2^ = 0.02.

Participants completed the memory questionnaire on three consecutive occasions. We developed a three‐phase paradigm, adapted from Brewer et al. [2018]; see also McCallum et al., [2016]. In Phase 1, participants freely generated the granularity of their responses to each question (i.e., FG or CG) without any instructions about grain size. In Phase 2, participants answered the same questions again but this time they were instructed to provide both an FG and a CG answer to each question (“forced report”), in a counterbalanced order. For example, when asked how many witnesses were in the bank, participants were asked to provide both an exact number (e.g., 3) and a range estimate (e.g., between 2 and 4). This allowed an examination of whether witnesses' reporting choices under the previous free report phase reflected the most accurate grain size available in their memory. Finally, in Phase 3, participants were asked to select one of their Phase 2 answers as their final answer. They were instructed to prioritize accuracy over level of detail by reporting CG options unless they were certain the FG detail was correct. In each phase, participants were not able to proceed to the next question without providing an answer to each question. They were instructed that if they were not sure or could not remember any of the answers to give their best answer, and not to say/type “do not know” or “cannot remember.” Participants provided judgments of confidence about the accuracy of each of their responses (0% very very unsure to 100% very very sure).

### *Coding*

Responses were coded by two independent raters for accuracy (correct or incorrect) and grain size (FG or CG). Following Weber and Brewer [2008], specific responses (e.g., sky blue; gray) were coded as FG while broader responses such as “dark” were coded as CG. Answers which included a phrase that suggested estimation, but ultimately included an FG response were coded as FG (e.g., “about/around 30 seconds” = 30 s, “grayish” = gray). Answers indirectly specifying a range were coded as CG (e.g., “20 seconds or under” = 0–20 s; “at least two but there could have been a third” = 2‐3, i.e., interpreted as a range), and ranges of 0 were coded as missing data (e.g., “2‐2”). Answers referring to the age of the car such as “very old” or “vintage old model” and those referring to an era (e.g., “1950s?”) were coded as CG. Color answers such as “multicolor” or “stripey” were coded as CG. Vague quantity‐related answers (e.g., “quite/lots/several”) and “do not know” responses were all coded as missing data. Where a participant gave two responses (e.g., “very dark/black,” “dark, almost black,” “brown or white,” or “late 1970s, maybe 1975?”), if they were two FG answers, the first answer was coded, and if they gave one FG and one CG response, the answer was coded as FG. In Phase 1, 25 items (1.98% of 1260 items) across seven participants were coded as missing data; in Phase 2, 18 items (0.71% of 2,520 items) across six participants were coded as missing; in Phase 3 one item was coded as missing (0.08% of 1,260 items). Strong agreement was reached between the two raters, with intraclass correlation coefficients of 0.99 for categorizing details as FG, 0.96 for categorizing CG details, and 0.98 for scoring accuracy (correct or incorrect).

## Results

### *Reporting Informativeness*

Informativeness scores for Phases 1 and 3 were calculated by dividing the number of FG details by the total number of details reported in that phase (i.e., FG/(FG + CG)). A 2 (Group: autistic, TD) × 2 (Delivery: social, online) × 2 (Phase: Phase 1 *free report*, Phase 3 *instructions to maximize accuracy*) mixed analysis of variance (ANOVA) was conducted with informativeness scores as the dependent variable. There was a main effect of Phase, with informativeness significantly dropping from Phase 1 to 3, *F*(1, 59) = 624.94, *P* < 0.001, *ηP* ^2^ = 0.91. Both groups demonstrated a strong preference for reporting FG details in Phase 1 (*M* informativeness = 0.94, *SD* = 0.09), which shifted to a preference for reporting more CG details in Phase 3 (*M* informativeness = 0.34, *SD* = 0.18). There was no effect of Group, *F*(1, 59) = 0.26, *P* = 0.612, *ηP* ^2^ = 0.004, Delivery, *F*(1, 59) = 0.24, *P* = 0.630, *ηP* ^2^ = 0.004, or Group × Delivery, *F*(1, 59) = 0.03, *P* = 0.872, *ηP* ^2^ < 0.001, Group × Phase, *F*(1, 59) = 3.20, *P* = 0.079, *ηP* ^2^ = 0.05, Delivery × Phase, *F*(1, 59) = 0.02, *P* = 0.888, *ηP* ^2^ < 0.001, or Group × Delivery × Phase interactions, *F*(1, 59) = 1.28, *P* = 0.262, *ηP* ^2^ = 0.02. That is, both groups showed a similar bias toward FG reporting, and the instructions to maximize accuracy had a similar effect in increasing CG reporting (reducing the informativeness of responses) for both groups, regardless of task delivery mode (i.e., social vs. online).

### *Reporting Accuracy*

Accuracy scores within each phase were calculated by the number of correct details as a function of total details reported (overall, and for FG and CG details separately). A series of Group × Delivery × Phase mixed ANOVAs were then conducted to examine whether autistic and TD witnesses differed in their spontaneous grain size reporting strategy and the impact of task instructions and delivery.^2^

#### *Strategic control under free report (phase 1 vs. phase 2)*

To examine whether autistic and TD participants differed in the effectiveness of their spontaneous grain size reporting strategy, overall accuracy scores in Phase 1 were compared with accuracy scores for FG and CG details in Phase 2 in two separate 2 (Group: autistic, TD) × 2 (Delivery: social, online) × 2 (Phase: Phase 1 *free report*, Phase 2 *forced report*) mixed ANOVAs for Phase 2 FG and Phase 2 CG accuracy, respectively. The first ANOVA indicated that Phase 1 overall accuracy was significantly higher than Phase 2 FG accuracy, *F*(1, 59) = 4.37, *P* = 0.041, *ηP* ^2^ = 0.07. There were no other main effects or interactions (all *P*s > 0.095, *ηP* ^2^s < 0.05).

The second ANOVA indicated that Phase 1 overall accuracy was significantly lower than Phase 2 CG accuracy, *F*(1, 59) = 76.47, *P* < 0.001, *ηP* ^2^ = 0.56. There was also a main effect of Delivery, *F*(1, 59) = 8.02, *P* = 0.006, *ηP* ^2^ = 0.12, whereby accuracy was significantly higher when the task was delivered socially compared to online. No other main effects or interactions were significant (*P*s > 0.281, *ηP* ^2^s < 0.02).

Thus, participants were exercising some grain size control (with more accurate free responses in Phase 1 than their forced FG responses in Phase 2). However, their Phase 1 responses were less accurate than their Phase 2 CG responses, indicating less than optimal control. The absence of group effects indicates that this was the case for both autistic and TD witnesses.

#### *Effect of instructions versus spontaneous reporting (phase 1 vs. phase 3)*

**Figure 1:**
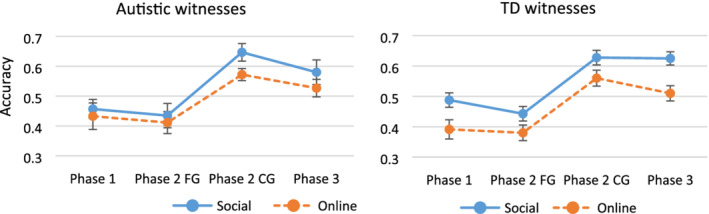
Accuracy at each Phase by Delivery condition for autistic and TD witnesses (error bars represent standard error of the means).

To examine the effect of instructions to maximize accuracy over informativeness compared to participants' spontaneous free reporting in Phase 1, a 2 (Group: autistic, TD) × 2 (Delivery: social, online) × 2 (Phase: Phase 1 *free report*, Phase 3 *instructions to maximize accuracy*) mixed ANOVA for overall accuracy was conducted. There was a main effect of Delivery, *F*(1, 59) = 7.72, *P* = 0.007, *ηP* ^2^ = 0.12, with higher accuracy for social (*M* = 0.52, *SD* = 0.10) compared to online delivery (*M* = 0.41, *SD* = 0.15). Accuracy was also significantly higher in Phase 3 (*M* = 0.56, *SD* = 0.12) compared to baseline free reporting in Phase 1 (*M* = 0.44, *SD* = 0.13), *F*(1, 59) = 44.39, *P* < 0.001, *ηP* ^2^ = 0.43. There was no effect of Group, *F*(1, 59) = 0.03, *P* = 0.862, *ηP* ^2^ = 0.001, or Group × Delivery, *F*(1, 59) = 1.67, *P* = 0.202, *ηP* ^2^ = 0.03, Group × Phase, *F*(1, 59) = 0.30, *P* = 0.589, *ηP* ^2^ = 0.01, Delivery × Phase, *F*(1, 59) = 0.46, *P* = 0.503, *ηP* ^2^ = 0.01, or Group × Delivery × Phase interactions, *F*(1, 59) = 0.02, *P* = 0.880, *ηP* ^2^ < 0.001. These data are displayed in Figure 1.

Thus, social question delivery and instructions to maximize accuracy were beneficial for both autistic and TD witnesses' accuracy.^3^

#### *Effect of instructions versus optimal reporting (phase 2 vs. phase 3)*

Two 2 (Group: autistic, TD) × 2 (Delivery: social, online) × 2 (Phase: Phase 2 *forced report*, Phase 3 *instructions to maximize accuracy*) mixed ANOVAs were conducted, comparing Phase 3 overall accuracy with Phase 2 FG and Phase 2 CG accuracy, respectively. The first ANOVA with Phase 2 FG details indicated a main effect of Delivery, with overall accuracy higher in the social than the online condition, *F*(1, 59) = 5.53, *P* = 0.022, *ηP* ^2^ = 0.09. There was also a main effect of Phase, with significantly higher overall accuracy in Phase 3 compared to Phase 2 FG responses, *F*(1,59) = 92.47, *P* < 0.001, *ηP* ^2^ = 0.61 (Fig. 1). No other main effects or interactions were significant (all *P*s > 0.173, *ηP* ^2^s < 0.03).

The second ANOVA with Phase 2 CG accuracy again showed a significant effect of Delivery with higher accuracy when the task was delivered socially compared to online, *F*(1, 59) = 8.81, *P* = 0.004, *ηP* ^2^ = 0.13. Phase 3 overall accuracy was again significantly lower than Phase 2 CG accuracy, *F*(1,59) = 23.02, *P* < 0.001, *ηP* ^2^ = 0.28. No other main effects or interactions were significant (all *P*s > 0.051, *ηP* ^2^s < 0.06).

Thus, both autistic and TD participants' accuracy was improved with the provision of instructions to maximize accuracy over informativeness and when questions were delivered socially, but grain size regulation was nevertheless still suboptimal for both groups. These data are displayed in Figure 1.

### *Metacognitive Monitoring*

#### *Confidence in accurate vs. inaccurate responses*

Metacognitive judgment accuracy was assessed through a series of 2 (Group: autistic, TD) × 2 (Delivery: social, online) × 2 (Accuracy: accurate, inaccurate) mixed ANOVAs with judgment of confidence ratings as the dependent variable for Phases 1, 2, and 3, respectively. There was a main effect of Accuracy, with significantly higher confidence for accurate than inaccurate answers (all *P*s < 0.001, *ηP* ^2^s > 0.48). There were no significant effects of Group (all *P*s > 0.466, *ηP* ^2^s < 0.01), Delivery (all *P*s > 0.223, *ηP* ^2^s < 0.03), or interaction effects (*P*s > 0.095, *ηP* ^2^s < 0.05). The mean number of correct and incorrect responses, and participants' mean confidence in these responses, are displayed in Table 2.

**Table 2:** Mean Number and Confidence of Accurately and Inaccurately Reported Details (*SD*s are in parentheses)

		Phase 1				Phase 2				Phase 3			
		Mean *N* accurate details	Mean Conf accurate	Mean *N* inaccurate details	Mean Conf inaccurate	Mean *N* accurate details	Mean Conf accurate	Mean *N* inaccurate details	Mean Conf inaccurate	Mean *N* accurate details	Mean Conf accurate	Mean *N* inaccurate details	Mean Conf inaccurate
Autistic	Social	9.13 (2.47)	62.66 (28.75)	10.73 (2.58)	49.40 (29.22)	21.60 (4.94)	66.19 (23.24)	18.33 (4.97)	53.19 (26.90)	11.6 (3.18)	70.55 (21.31)	8.40 (3.18)	66.29 (24.58)
	Online	7.60 (2.32)	60.83 (28.28)	10.60 (4.15)	46.86 (25.28)	19.13 (3.36)	62.34 (30.36)	19.33 (3.92)	49.31 (26.03)	10.53 (2.26)	65.86 (30.17)	9.47 (2.26)	56.48 (26.29)
TD	Social	9.72 (2.02)	64.39 (24.76)	10.22 (2.05)	44.20 (23.72)	21.33 (3.33)	69.64 (22.49)	18.50 (3.37)	49.16 (23.84)	12.50 (1.89)	72.04 (21.80)	7.44 (1.89)	60.05 (20.86)
	Online	7.73 (2.40)	56.64 (24.69)	11.87 (2.45)	45.91 (19.35)	18.80 (3.08)	61.18 (20.96)	21.20, (3.08	50.34 (23.82)	10.20 (1.93)	64.52 (21.67)	9.80 (1.93)	58.48 (25.61)

#### *Confidence–accuracy calibration*

**Figure 2:**
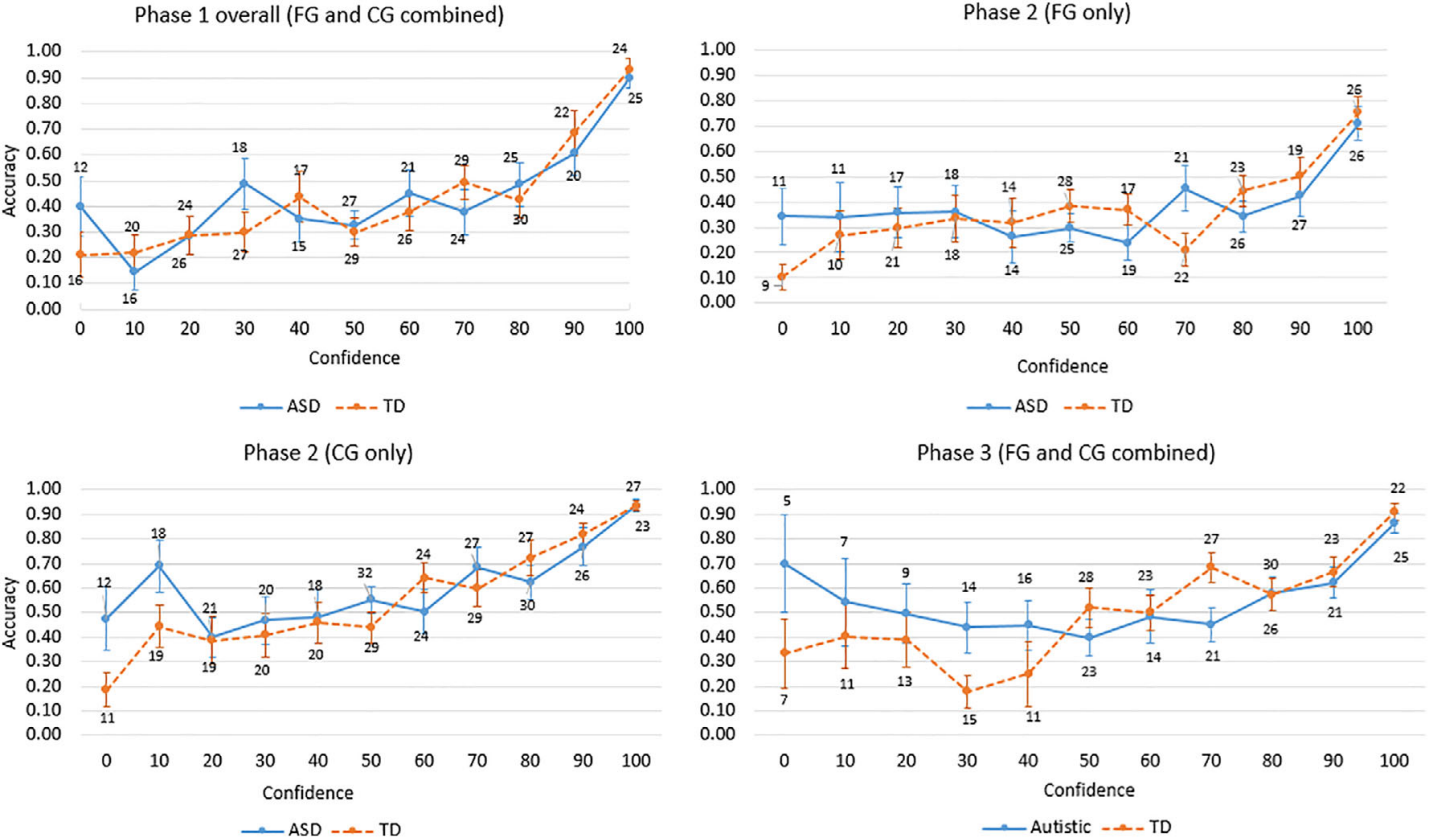
Confidence–accuracy calibration curves for autistic and TD witnesses within each Phase. The frequency of judgments in each confidence category is presented with each data point.

Figure 2 displays confidence–accuracy calibration curves for autistic and TD witnesses, representing the overall correspondence between their assessed and actual probabilities of being correct in each phase (with separate curves for FG and CG details in Phase 2). All curves display a generally positive relationship, indicative of confidence and accuracy being calibrated for both groups. However, for both groups, there was a sharp drop from around 90% accuracy at 100% confidence under free report in Phase 1, to around 70% accuracy at 100% confidence and 45% accuracy at 90% confidence when participants were forced to provide FG responses in Phase 2. In other words, rather than simply lowering their judgments of confidence when pressured to provide (inaccurate) FG responses, participants instead reported inaccurate items with high confidence, indicating that confidence is less diagnostic under conditions where witnesses feel pressured to be informative.

#### *Gamma analyses*

Kruskal–Goodman Gamma correlations [see Nelson, 1984] were calculated for each individual participant between the assessed probability correct for each answer and whether or not the answer actually was correct (i.e., the total number of accurate and inaccurate responses at each level of confidence: i.e., 0%–100% at 10% increments). Gamma correlations range between +1 and −1, with a score of 0 indicating chance‐level accuracy. A large positive gamma value indicates high correspondence between confidence in the correctness of one's answers and their actual correctness, while a large negative value indicates that confidence judgments were contrariwise to recall performance (i.e., below chance performance).

Gamma scores could not be calculated for one participant in Phase 3 as they did not report a large enough range of confidence choices, and tests of normal distribution revealed an outlier in the TD group. These cases were removed and a series of one‐sample *t*‐tests within each Group, Delivery, and Phase combination indicated that gamma scores were significantly greater than chance (i.e., 0) across all conditions (all *P*s < 0.002, *d*s > 1.04). A 2 (Group) × 2 (Delivery) × 3 (Phase) mixed ANOVA was then conducted with Gamma scores as the dependent variable. There were no effects of Group, *F*(1,57) = 0.36, *P* = 0.552, *ηP* ^2^ = 0.01, Delivery, *F*(1,57) = 0.02, *P* = 0.884, *ηP* ^2^ < 0.001, or Group × Delivery, *F*(1, 57) = 3.15, *P* = 0.081, *ηP* ^2^ = 0.05, Group × Phase, *F*(2, 114) = 1.05, *P* = 0.353, *ηP* ^2^ = 0.02, Delivery × Phase, *F*(2, 114) = 0.73, *P* = 0.484, *ηP* ^2^ = 0.01, or Group × Delivery × Phase interactions, *F*(2,114) = 0.34, *P* = 0.715, *ηP* ^2^ = 0.01. (Table 3).

**Table 3:** Mean Gamma Coefficients for Autistic and TD Witnesses Within Social and Online Delivery Conditions (*SD*s are in Parentheses)

	Phase 1		Phase 2		Phase 3	
	Social delivery	Online delivery	Social delivery	Online delivery	Social delivery	Online delivery
Autistic	0.40 (0.29)	0.57 (0.20)	0.38 (0.16)	0.41 (0.23)	0.31 (0.24)	0.38 (0.36)
TD	0.49 (0.23)	0.44 (0.23)	0.45 (0.20)	0.32 (0.23)	0.47 (0.29)	0.31 (0.37)

### *Metacognitive Control*

**Figure 3:**
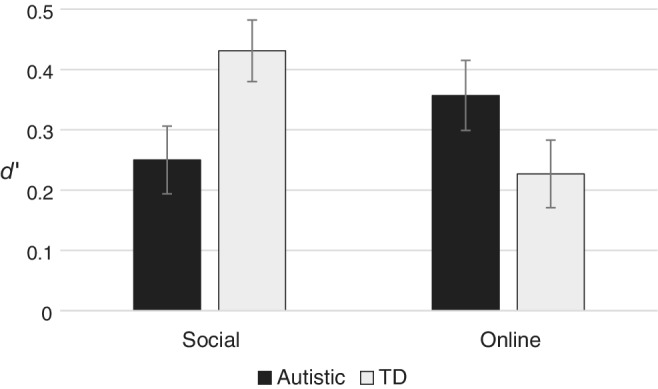
*d′* scores (metacognitive control over reporting) for autistic and TD participants in social and online delivery conditions (error bars reflect standard error of the mean).

Following Grainger et al. [2016] and Sawyer et al. [2014], d‐prime (*d′*) was calculated using participants' hit rates (HR) and false alarm rates (FAR) to examine strategic control effectiveness. HR was the number of hits (correct Phase 2 FG responses that were put forward in Phase 3) plus the number of correct rejections (CG responses that were put forward in Phase 3 where Phase 2 FG responses were incorrect), divided by the total number of responses. FAR was the number of false alarms (incorrect Phase 2 FG responses that were put forward in Phase 3) plus the number of misses (CG responses put forward in Phase 3 where Phase 2 FG responses had been correct), divided by the total number of responses. *d*′ was then calculated as the difference between HR and FAR. A *d*′ score of 0 indicates no difference between HR and FAR and thus ineffective control, while *d*′ scores significantly above 0 indicate higher HRs than FARs and thus effective control over reporting decisions. A 2 (Group) × 2 (Delivery) ANOVA with *d*′ as the dependent variable indicated no significant main effects of Group, *F*(1, 58) = 0.21, *P* = 0.651, *ηP* ^2^ = 0.004, or Delivery, *F*(1, 58) = 0.77, *P* = 0.384, *ηP* ^2^ = 0.01, but a significant Group × Delivery interaction, *F*(1, 58) = 7.96, *P* = 0.007, *ηP* ^2^ = 0.12. As shown in Figure 3, TD witnesses demonstrated significantly better reporting control than autistic witnesses under social delivery (*P* = 0.003), but there was no significant difference between groups when using online delivery (*P* = 0.195).

## Discussion

Although autistic individuals have been reported to show subtle difficulties in monitoring and/or controlling their reporting accuracy [e.g., Brosnan et al., 2016; Cherkaoui & Gilbert, 2017; Grainger et al., 2014, 2016; Williams et al., 2018], this has not been previously examined in the context of episodic memory. This gap is important because diminished monitoring and control processes may contribute to the often‐reported difficulties in episodic event memory in autism [e.g., when providing an eyewitness account; see Maras, 2020]. Contrary to predictions, autistic witnesses did not show impaired metacognitive monitoring or control relative to TD witnesses. Indeed, both autistic and TD witnesses showed a similarly strong preference for reporting (less accurate) FG detail under free report conditions, and both groups benefitted from explicit task instructions with improved (although still suboptimal) accuracy when instructed to maximize accuracy over informativeness. There was, however, one important caveat: while delivering the questions socially improved overall accuracy rates for both groups compared to online delivery, the autistic group nevertheless showed poorer metacognitive control in their reporting decisions compared to TD witnesses under social, but not online, reporting conditions.

At first glance, the undiminished episodic memory demonstrated by autistic participants in the current study is surprising, given the substantial existing literature documenting episodic memory impairments in this group [see e.g., Boucher et al., 2012; Boucher & Bowler, 2008]. However, memory difficulties in autism are not pervasive (particularly in individuals of average or above average levels of intelligence) and tend to depend on the nature of the task. Specifically, autistic people's difficulties are particularly marked on tests requiring a free narrative account [e.g., Bowler, Gaigg, & Gardiner, 2008; Bowler, Matthews, & Gardiner, 1997], while differences between groups often diminish once tests of cued recall or recognition are used [e.g., Almeida, Lamb, & Weisblatt, 2019; Bowler et al., 1997, 2008; see also Maras, 2020]. Such findings have led Bowler and colleagues to propose the task support hypothesis [Bowler et al., 1997; Bowler et al., 2004], which posits that memory performance in autism is improved on tasks that provide more support for the to‐be‐remembered material at test. Thus, the structured paradigm used in the present study might explain the observed intact reporting accuracy of autistic participants. Findings also suggest that the task support hypothesis may further apply to metacognitive processes, in line with previous findings that autistic individuals are able to monitor and regulate their performance strategies when external cues to aid recall are available to them [e.g., Farrant, Boucher, & Blades, 1999; see also Sawyer et al., 2014].

We used cued recall questions in order to generate specific responses that were readily codable for grain size in an objective and comparable way between participants, in line with previous grain size research with TD witnesses [e.g., McCallum et al., 2016, 2019; Weber & Brewer, 2008]. However, it is important for future research to extend these findings using more unsupported tests (such as a free narrative account) to confirm the absence of difficulties in metacognitive monitoring and control by autistic people in episodic memory reporting. A potential approach to this might be to generate a free narrative account from participants in Phase 1 before presenting their account back to them in Phase 2 and obtaining judgments of confidence for each unit of information reported (coding these, where possible, for grain size). Brewer et al. [2018] examined grain size reporting across tests of both free recall and cued recall with TD witnesses and found they rarely reported CG details under open‐ended interview conditions, but they did so under cued recall forced‐report conditions. That is, the cued recall forced‐report procedure provided a means by which accessible, but otherwise unreported, CG information was provided. Thus, a free recall paradigm may provide a more sensitive measure of participants' spontaneous grain size control and may be more likely to reveal any latent differences between autistic and TD groups.

As an aside, it is worth noting that both autistic and TD witnesses' accuracy was only around 75% at the 90%–100% confidence level when they were forced to provide FG responses in Phase 2, which is inconsistent with the high free report confidence–accuracy observed in Phase 1, and with Wixted, Mickes, and Fisher's [2018] view that accuracy for very high confidence (i.e., 90%–100%) responses is extremely high. Thus, when under pressure, autistic and TD witnesses appear to produce a greater proportion of inaccurate items at high confidence, indicating that confidence is less diagnostic of accuracy when witnesses are pressured to be informative [see also Brewer et al., 2018]. Here the pressure was explicit, but of course, in actual police investigations, contextual factors could lead to witnesses perceiving a need to be informative.

The instruction to maximize accuracy over informativeness improved both autistic and TD witnesses' reporting accuracy similarly and substantially. It is important to note, however, that participants underwent an interim forced report procedure to access both FG and CG detail accuracy in Phase 2. We cannot rule out the possibility that the explicit instructions to generate a CG response in Phase 2 increased the perceived appropriateness of this option to drive the greater CG responding in Phase 3. Alternatively, the process of having previously generated both FG and CG responses may alter the way participants retrieve and evaluate candidate responses in the subsequent phase [Sauer & Hope, 2016]. Notwithstanding this, that the autistic group also apparently benefited from the explicit instruction to prioritize accuracy is consistent with the contention that autistic individuals' performance is more impaired the greater the degree of open‐endedness of the test situation [e.g., Ciesielski & Harris, 1997; Van Eylen et al., 2011; White, 2013; White et al., 2009], and has implications for the instructions they receive about the importance of accuracy when providing eyewitness testimony.

Witnesses were more accurate in their recall when questions were delivered socially rather than online. This increase in accuracy was not accompanied by a reduction in informativeness, indicating that other processes, such as increased motivation and feelings of accountability, were involved [e.g., McCallum et al., 2016; Vandierendonck & Van Damme, 1988; but see Taylor & Dando, 2018]. That the social condition improved accuracy rates similarly for autistic and TD witnesses is in contrast to the view that autism is marked by diminished social motivation [Chevallier et al., 2012] and is more in line with recent suggestions that autistic people *are* socially motivated, but that social‐cognitive difficulties can limit their ability to read and respond appropriately to social cues [see e.g., Hull et al., 2017; Jaswal & Akhtar, 2018; Livingston, Shah, & Happé, 2018]. Indeed, despite this improvement in overall accuracy, the autistic group showed poorer reporting control than TD witnesses in the social condition, but this difference between groups dissipated when the task was delivered online. Thus, it seems that while autistic participants may be as motivated as TD participants to enhance their accuracy under social reporting conditions, underlying social‐cognitive difficulties may over‐burden autistic individuals' executive resources in contexts involving social interaction, limiting their ability to make optimal reporting decisions [see also Dichter & Belger, 2007].

The current study has a number of important practical implications for obtaining eyewitness testimony from autistic individuals. First, findings highlight that autistic witnesses can provide as detailed and accurate testimony as non‐autistic witnesses when specific and cued questions are used [see Maras, 2020, for a detailed discussion of the implications of this for practice]. Second, autistic witnesses showed a similarly strong tendency as TD witnesses to report more FG detail (at the expense of accuracy), but both groups shifted toward reporting more accurate but coarser‐grained information when instructed to do so. Thus, when deciding on an appropriate questioning strategy to elicit an eyewitness account, investigators need to determine whether greater potential investigative leads (i.e., FG but potentially less accurate information) or preserving the integrity of the information that is reported (i.e., with more accurate but CG detail) is more important. Third, while both groups were more accurate in their recall of events when questions were delivered socially, the autistic group showed a subtle impairment in reporting control under this condition, indicating that social situations may be motivating but nevertheless more cognitively burdensome for autistic witnesses. Further research is needed using different reporting conditions, but tentatively this finding suggests that interview situations, which are fundamentally social (e.g., where questions are delivered by an interviewer) but in which social complexities and ambiguities have been minimized (e.g., avoiding the pressure for eye contact, using more direct language, etc.) may optimize recall from autistic witnesses [see also Hsu & Teoh, 2017]. Finally, the finding that both autistic and TD witnesses produced a greater proportion of inaccurate items at high confidence under forced‐report conditions highlights the importance of minimizing investigator pressure and explicitly offering the option for witnesses to say if they “do not know” or “cannot remember” [see Bull, 2010).

It is important to acknowledge the limitations of the current study. We cannot rule out the possibility that null effects such as the lack of between‐group differences in reporting accuracy were due to insufficient power to detect smaller effects. However, all effect sizes for between‐groups comparisons were very small (all *ηP* ^2^s < 0.01), suggesting that any group differences in this regard were unlikely to be meaningful on a practical level. It is also worth reiterating that the absence of group differences can be readily accounted for by the structured nature of the task with specific cued questions, which likely offered sufficient task support to diminish episodic memory differences between groups (see Bowler et al., 1997, Bowler et al., 2004). It is a further limitation that, although the participants in the current study were matched on age and IQ, it was not possible to match groups on sex, which may be pertinent given that some sex differences have been reported in episodic memory (see Herlitz & Rehnman, 2008]. When sex differences are found, however, they tend to favor females (Herlitz & Rehnman, 2008]. Since the autistic group in the current study comprised a greater ratio of males to females this should, if anything, have made detection of autistic impairment more likely, yet no such differences were found.

To conclude, to our knowledge, this is the first study to explore metacognitive monitoring and control processes surrounding confidence in episodic memories in an eyewitness testimony context. We found no evidence of autistic impairment in episodic memory or metacognitive monitoring and control processes overall. However, social delivery of the task appeared to negatively impact autistic witnesses' ability to optimally control their reporting decisions in terms of withholding details that were incorrect and putting forward accurate details at the finest level of grain size available in memory. These difficulties were not present when the task was delivered online. Future research should extend these findings using free recall rather than cued questions. Findings have practical implications for the reliability of autistic eyewitness evidence and the format in which they are questioned.

## Supporting information


**Appendix** **1**. Phase 1 (free report) instructions and questions

## Endnote

1 From here on the term “witness” is used throughout to encompass both third party witnesses and victims who, through providing evidence in the Criminal Justice System, become a witness.

2 Separate ANOVAs, rather than a single omnibus ANOVA, were conducted because in Phase 2 respondents provided both FG and CG answers.

3 It is worth noting, however, that a subsidiary analysis of confidence–accuracy with Phase 3 FG data nevertheless suggested that both groups found it hard to follow this instruction, with 88% of items reported at FG being rated at 90% or less confidence.
