# Supplementary material for: Metacognitive Monitoring and Control of Eyewitness Memory Reports in Autism
Source: Autism Res. 2020 Feb 13;13(11):2017–29. doi: 10.1002/aur.2278 (PMC7754119; doi:10.1002/aur.2278)
Supplement: Supplementary file 1 — Appendix 1. Phase 1 (free report) instructions and questions [file AUR-13-2017-s001.docx]

**Appendix 1. Phase 1 (free report) instructions and questions**

**Phase 1 instructions – please read [listen] carefully**

 Please answer ALL of the following questions about the video you saw

After each answer there is a confidence scale going from 0% (very very unsure) to 100% (very very sure). Please rate how sure you are that the response you provided is correct for each answer.

There are 3 Phases, each with 20 questions.

If you are not sure or can’t remember, just give the best answer you can (please do not answer with ‘I don’t know’ or ‘can’t remember’).

Any questions?

**1. What was the colour of the beanie worn by the robber NEAR THE DOOR?**

How sure are you that your response is correct?

0% 10% 20% 30% 40% 50% 60% 70% 80% 90% 100%

**2. What was the colour of the jacket worn by the robber NEAR THE DOOR?**

How sure are you that your response is correct?

0% 10% 20% 30% 40% 50% 60% 70% 80% 90% 100%

**3. What was the colour of the top worn by the robber NEAR THE DOOR?**

How sure are you that your response is correct?

0% 10% 20% 30% 40% 50% 60% 70% 80% 90% 100%

**4. What was the colour of the trousers worn by the robber NEAR THE DOOR?**

How sure are you that your response is correct?

0% 10% 20% 30% 40% 50% 60% 70% 80% 90% 100%

**5. What was the main colour of the bag carried by the robber NEAR THE DOOR?**

How sure are you that your response is correct?

0% 10% 20% 30% 40% 50% 60% 70% 80% 90% 100%

**6. What was the main colour of the hat worn by the robber AT THE COUNTER?**

How sure are you that your response is correct?

0% 10% 20% 30% 40% 50% 60% 70% 80% 90% 100%

**7. What was the main colour of the jacket worn by the robber AT THE COUNTER?**

How sure are you that your response is correct?

0% 10% 20% 30% 40% 50% 60% 70% 80% 90% 100%

**8. What was the eye colour of the robber AT THE COUNTER?**

How sure are you that your response is correct?

0% 10% 20% 30% 40% 50% 60% 70% 80% 90% 100%

**9. What was the hair colour of the robber AT THE COUNTER?**

How sure are you that your response is correct?

0% 10% 20% 30% 40% 50% 60% 70% 80% 90% 100%

**10. What was the colour of the t-shirt worn by the robber AT THE COUNTER?**

How sure are you that your response is correct?

0% 10% 20% 30% 40% 50% 60% 70% 80% 90% 100%

**11. What was the main colour of the shirt worn by the robber AT THE COUNTER?**

How sure are you that your response is correct?

0% 10% 20% 30% 40% 50% 60% 70% 80% 90% 100%

**12. What was the main colour of the bag carried by the robber AT THE COUNTER?**

How sure are you that your response is correct?

0% 10% 20% 30% 40% 50% 60% 70% 80% 90% 100%

**13. How long did the robbery take to occur from the time you saw the robbers until they drove away?**

How sure are you that your response is correct?

0% 10% 20% 30% 40% 50% 60% 70% 80% 90% 100%

**14. How many robbers were in the video?**

How sure are you that your response is correct?

0% 10% 20% 30% 40% 50% 60% 70% 80% 90% 100%

**15. How many witnesses were in the bank?**

How sure are you that your response is correct?

0% 10% 20% 30% 40% 50% 60% 70% 80% 90% 100%

**16. How many times did the robber AT THE COUNTER talk?**

How sure are you that your response is correct?

0% 10% 20% 30% 40% 50% 60% 70% 80% 90% 100%

**17. How long did it take the robbers to run from the bank to the car?**

How sure are you that your response is correct?

0% 10% 20% 30% 40% 50% 60% 70% 80% 90% 100%

**18. What colour was the car?**

How sure are you that your response is correct?

0% 10% 20% 30% 40% 50% 60% 70% 80% 90% 100%

**19. How many doors did the car have?**

How sure are you that your response is correct?

0% 10% 20% 30% 40% 50% 60% 70% 80% 90% 100%

**20. How old was the car?**

How sure are you that your response is correct?

0% 10% 20% 30% 40% 50% 60% 70% 80% 90% 100%

**Appendix 2. Phase 2 (forced report) instructions and questions**

**Phase 2 instructions - please read [listen] carefully**

**For each question in this phase you will be asked to give two responses**

One answer will be a very specific detail – for example “sky blue” as a response to “what colour?”

The other answer will be a broader detail – for example “light” as a response to “what colour?”

**Make sure you read [listen to] each question carefully because the two types of responses won’t be in the same order for each question**

**Here are some example questions and answers:**

*What colour were the walls in the bank?*

Exact colour **white**

How sure are you that your response is correct?

0% 10% **20%** 30% 40% 50% 60% 70% 80% 90% 100%

General colour (light, medium, dark) **light**

How sure are you that your response is correct?

0% 10% 20% 30% 40% 50% 60% 70% **80%** 90% 100%

*How many people were in the bank during the robbery?*

Between (range) **4** and **8**

How sure are you that your response is correct?

0% 10% 20% 30% 40% **50%** 60% 70% 80% 90% 100%

Exact **6**

How sure are you that your response is correct?

0% **10%** 20% 30% 40% 50% 60% 70% 80% 90% 100%

**1. What was the colour of the beanie worn by the robber NEAR THE DOOR?**

General colour (light, medium, dark) _______________________

How sure are you that your response is correct?

0% 10% 20% 30% 40% 50% 60% 70% 80% 90% 100%

Exact colour __________________________________________

How sure are you that your response is correct?

0% 10% 20% 30% 40% 50% 60% 70% 80% 90% 100%

**2. What was the colour of the jacket worn by the robber NEAR THE DOOR?**

Exact colour __________________________________________

How sure are you that your response is correct?

0% 10% 20% 30% 40% 50% 60% 70% 80% 90% 100%

General colour (light, medium, dark) _______________________

How sure are you that your response is correct?

0% 10% 20% 30% 40% 50% 60% 70% 80% 90% 100%

**3. What was the colour of the top worn by the robber NEAR THE DOOR?**

Exact colour __________________________________________

How sure are you that your response is correct?

0% 10% 20% 30% 40% 50% 60% 70% 80% 90% 100%

General colour (light, medium, dark) _______________________

How sure are you that your response is correct?

0% 10% 20% 30% 40% 50% 60% 70% 80% 90% 100%

**4. What was the colour of the trousers worn by the robber NEAR THE DOOR?**

General colour (light, medium, dark) _______________________

How sure are you that your response is correct?

0% 10% 20% 30% 40% 50% 60% 70% 80% 90% 100%

Exact colour __________________________________________

How sure are you that your response is correct?

0% 10% 20% 30% 40% 50% 60% 70% 80% 90% 100%

**5. What was the main colour of the bag carried by the robber NEAR THE DOOR?**

General colour (light, medium, dark) _______________________

How sure are you that your response is correct?

0% 10% 20% 30% 40% 50% 60% 70% 80% 90% 100%

Exact colour __________________________________________

How sure are you that your response is correct?

0% 10% 20% 30% 40% 50% 60% 70% 80% 90% 100%

**6. What was the main colour of the hat worn by the robber AT THE COUNTER?**

Exact colour __________________________________________

How sure are you that your response is correct?

0% 10% 20% 30% 40% 50% 60% 70% 80% 90% 100%

General colour (light, medium, dark) _______________________

How sure are you that your response is correct?

0% 10% 20% 30% 40% 50% 60% 70% 80% 90% 100%

**7. What was the main colour of the jacket worn by the robber AT THE COUNTER?**

Exact colour __________________________________________

How sure are you that your response is correct?

0% 10% 20% 30% 40% 50% 60% 70% 80% 90% 100%

General colour (light, medium, dark) _______________________

How sure are you that your response is correct?

0% 10% 20% 30% 40% 50% 60% 70% 80% 90% 100%

**8. What was the eye colour of the robber AT THE COUNTER?**

General colour (light, medium, dark) _______________________

How sure are you that your response is correct?

0% 10% 20% 30% 40% 50% 60% 70% 80% 90% 100%

Exact colour __________________________________________

How sure are you that your response is correct?

0% 10% 20% 30% 40% 50% 60% 70% 80% 90% 100%

**9. What was the hair colour of the robber AT THE COUNTER?**

General colour (light, medium, dark) _______________________

How sure are you that your response is correct?

0% 10% 20% 30% 40% 50% 60% 70% 80% 90% 100%

Exact colour __________________________________________

How sure are you that your response is correct?

0% 10% 20% 30% 40% 50% 60% 70% 80% 90% 100%

**10. What was the colour of the t-shirt worn by the robber AT THE COUNTER?**

Exact colour __________________________________________

How sure are you that your response is correct?

0% 10% 20% 30% 40% 50% 60% 70% 80% 90% 100%

General colour (light, medium, dark) _______________________

How sure are you that your response is correct?

0% 10% 20% 30% 40% 50% 60% 70% 80% 90% 100%

**11. What was the main colour of the shirt worn by the robber AT THE COUNTER?**

Exact colour __________________________________________

How sure are you that your response is correct?

0% 10% 20% 30% 40% 50% 60% 70% 80% 90% 100%

General colour (light, medium, dark) _______________________

How sure are you that your response is correct?

0% 10% 20% 30% 40% 50% 60% 70% 80% 90% 100%

**12. What was the main colour of the bag carried by the robber AT THE COUNTER?**

General colour (light, medium, dark) _______________________

How sure are you that your response is correct?

0% 10% 20% 30% 40% 50% 60% 70% 80% 90% 100%

Exact colour __________________________________________

How sure are you that your response is correct?

0% 10% 20% 30% 40% 50% 60% 70% 80% 90% 100%

**13. How long did the robbery take to occur from the time you saw the robbers until they drove away?**

Between _______________ and ___________________ (range of seconds)

How sure are you that your response is correct?

0% 10% 20% 30% 40% 50% 60% 70% 80% 90% 100%

Exact number of seconds __________________________

How sure are you that your response is correct?

0% 10% 20% 30% 40% 50% 60% 70% 80% 90% 100%

**14. How many robbers were in the video?**

Exact number ____________________________________

How sure are you that your response is correct?

0% 10% 20% 30% 40% 50% 60% 70% 80% 90% 100%

Between _______________ and ___________________ (range)

How sure are you that your response is correct?

0% 10% 20% 30% 40% 50% 60% 70% 80% 90% 100%

**15. How many witnesses were in the bank?**

Exact number ____________________________________

How sure are you that your response is correct?

0% 10% 20% 30% 40% 50% 60% 70% 80% 90% 100%

Between _______________ and ___________________ (range)

How sure are you that your response is correct?

0% 10% 20% 30% 40% 50% 60% 70% 80% 90% 100%

**16. How many times did the robber AT THE COUNTER talk?**

Between _______________ and ___________________ (range)

How sure are you that your response is correct?

0% 10% 20% 30% 40% 50% 60% 70% 80% 90% 100%

Exact number ____________________________________

How sure are you that your response is correct?

0% 10% 20% 30% 40% 50% 60% 70% 80% 90% 100%

**17. How long did it take the robbers to run from the bank to the car?**

Between _______________ and ___________________ (range of seconds)

How sure are you that your response is correct?

0% 10% 20% 30% 40% 50% 60% 70% 80% 90% 100%

Exact number of seconds __________________________

How sure are you that your response is correct?

0% 10% 20% 30% 40% 50% 60% 70% 80% 90% 100%

**18. What colour was the car?**

Exact colour __________________________________________

How sure are you that your response is correct?

0% 10% 20% 30% 40% 50% 60% 70% 80% 90% 100%

General colour (light, medium, dark) _______________________

How sure are you that your response is correct?

0% 10% 20% 30% 40% 50% 60% 70% 80% 90% 100%

**19. How many doors did the car have?**

Exact number ____________________________________

How sure are you that your response is correct?

0% 10% 20% 30% 40% 50% 60% 70% 80% 90% 100%

Between _______________ and ___________________ (range)

How sure are you that your response is correct?

0% 10% 20% 30% 40% 50% 60% 70% 80% 90% 100%

**20. How old was the car?**

Between _______________ and ___________________ (range of years)

How sure are you that your response is correct?

0% 10% 20% 30% 40% 50% 60% 70% 80% 90% 100%

Exact number of years __________________________

How sure are you that your response is correct?

0% 10% 20% 30% 40% 50% 60% 70% 80% 90% 100%

**Appendix 3. Phase 3 (instructions to maximise accuracy) instructions and example question**

**Phase 3 Instructions – Please read [listen] carefully**

You will be shown [reminded of] the two responses you gave for each question in Phase 2. Please pick ONLY ONE of these answers as your final answer.

We want you to try your best to maximise **ACCURACY** over**INFORMATIVENESS**. This means that you should report exact answers (e.g., that the tiles on the floor of the bathroom were **navy blue**) only if you are certain they are accurate; if you think that the general or broader answer (e.g., that the tiles on the floor of the bathroom were **dark-coloured**) is more accurate then please select that.

Try to pick the answer you feel is most likely to be accurate each time, as well as choosing [telling me] how sure you are that your responses are correct.

 If you are not sure or can’t remember just give the best answer you can (please do not type ‘don’t know’ or ‘can’t remember’).

**1. What was the colour of the beanie worn by the robber NEAR THE DOOR (select one as your final answer)?**

General colour___________ **light**______________ (light, medium, dark)

Exact colour ______ **sky blue**________________________

How sure are you that your response is correct?

0% 10% 20% 30% 40% 50% 60% 70% 80% 90% 100%
